# P108 Central nervous system lymphomatoid granulomatosis and toxoplasmosis in a patient with systemic lupus erythematosus and Sjögren disease: a diagnostic challenge

**DOI:** 10.1093/rap/rkag113.138

**Published:** 2026-11-03

**Authors:** Elham Abdellatif, Iman Ali

**Affiliations:** Gloucestershire Hospitals NHS Foundation Trust, Gloucester, United Kingdom; Gloucestershire Hospitals NHS Foundation Trust, Gloucester, United Kingdom

## Abstract

**Introduction:**

**Introduction:** Lymphomatoid granulomatosis (LYG) is a rare Epstein-Barr virus (EBV)-driven lymphoproliferative disorder characterized by angiocentric and angiodestructive infiltration of EBV-positive B cells within a polymorphous inflammatory background. Pulmonary involvement is typical; however, isolated central nervous system (CNS) disease is exceptionally uncommon and presents a significant diagnostic challenge, particularly in immunocompromised patients.

**Case description:**

**Case presentation:** 62-year-old woman with systemic lupus erythematosus (SLE) diagnosed in 1992 and biopsy-proven class V lupus nephritis (2004) presented with a 2-week history of expressive dysphagia on a background of two months of progressive word- finding difficulty, worsening ataxia and intermittent fever exceeding 38C. she also had secondary Sjögren’s syndrome (1995). Her medications included mycophenolate mofetil (1.5 g morning, 1 g evening), prednisone 5 mg daily, and hydroxychloroquine 400/200 mg on alternate days.

She reported recurrent chest infections requiring multiple antibiotic courses, including one following a cat scratch several months prior, raising concern for opportunistic infection. She denied headache, visual disturbance, chest pain, urinary symptoms, or bowel disturbance. There were no clinical features suggestive of active lupus flare.

Laboratory investigations showed normal liver and renal function, normal inflammatory markers and stable full blood count. Cerebrospinal fluid (CSF) analysis demonstrated lymphocytic pleocytosis (WBC count 59cells/**μl**, 100%lymphocytes).

MRI brain revealed multifocal supratentorial and infratentorial enhancing lesions, including the right cerebellum. CT thorax, abdomen and pelvis showed no systemic malignancy or vasculitis.

Extensive infectious screening was negative except for positive toxoplasma IgM serology, although CSF PCR was negative. Empirical pyrimethamine and atovaquone were initiated.

Cerebellar biopsy confirmed grade 3 lymphomatoid granulomatosis with an EBV viral load of 120 copies/mL.

Mycophenolate mofetil and hydroxychloroquine were discontinued. Treatment with high-dose methotrexate combined with rituximab was initiated.

Follow-up MRI after the first chemotherapy cycle demonstrated a partial response but new enhancing lesions, raising uncertainty between progressive LYG and toxoplasmosis. Multidisciplinary discussion prompted escalation of methotrexate and repeat CSF and blood PCR testing.

After the second cycle, EBV viral load fell to < 50 copies/ mL and MRI demonstrated radiological stability with resolution of several lesions. CSF PCR for EBV and toxoplasma was negative. Planned management included 5 cycles of rituximab -methotrexate with ongoing monitoring.

**Discussion:**

**Discussion:** Neurological symptoms and SLE frequently raise concern for neuropsychiatric lupus, infection, or cerebral vasculitis. In immunosuppressed patients, however, EBV-driven lymphoproliferative disorders must also be considered. This case illustrates the diagnostic complexity created by overlapping infectious and malignant processes.

Patients with SLE demonstrated impaired immune surveillance and increased EBV reactivation, which may contribute to lymphoproliferative disease development. Chronic immunosuppression further increases the risk. Isolated CNS lymphomatoid granulomatosis is rare and often radiologically indistinguishable from infection or inflammatory disease.

Positive toxoplasma serology initiated supported an infectious diagnosis, delaying recognition of malignancy. Definitive diagnosis required a brain biopsy, highlighting the importance of early tissue sampling when clinical progression or imaging findings remain unexplained.

Multidisciplinary collaboration between rheumatology, neurology, infectious diseases, and haematology was critical to successful management

**Key learning points:**

**Conclusion**: This case highlights the importance of considering EBV-associated lymphoproliferative disorders, including lymphomatoid granulomatosis in patients with SLE presenting with atypical neurological features and suspicious MRI findings.

